# From Beef to Bees: High-Throughput Kinome Analysis to Understand Host Responses of Livestock Species to Infectious Diseases and Industry-Associated Stress

**DOI:** 10.3389/fimmu.2020.00765

**Published:** 2020-05-15

**Authors:** Antonio Facciuolo, Connor Denomy, Sean Lipsit, Anthony Kusalik, Scott Napper

**Affiliations:** ^1^Vaccine and Infectious Disease Organization - International Vaccine Centre, University of Saskatchewan, Saskatoon, SK, Canada; ^2^Department of Computer Science, University of Saskatchewan, Saskatoon, SK, Canada; ^3^Department of Biochemistry, Microbiology and Immunology, University of Saskatchewan, Saskatoon, SK, Canada

**Keywords:** kinome, kinase, phosphorylation, peptide array, stress, infectious disease

## Abstract

Within human health research, the remarkable utility of kinase inhibitors as therapeutics has motivated efforts to understand biology at the level of global cellular kinase activity (the kinome). In contrast, the diminished potential for using kinase inhibitors in food animals has dampened efforts to translate this research approach to livestock species. This, in our opinion, was a lost opportunity for livestock researchers given the unique potential of kinome analysis to offer insight into complex biology. To remedy this situation, our lab developed user-friendly, cost-effective approaches for kinome analysis that can be readily incorporated into most research programs but with a specific priority to enable the technology to livestock researchers. These contributions include the development of custom software programs for the creation of species-specific kinome arrays as well as comprehensive deconvolution and analysis of kinome array data. Presented in this review are examples of the application of kinome analysis to highlight the utility of the technology to further our understanding of two key complex biological events of priority to the livestock industry: host immune responses to infectious diseases and animal stress responses. These advances and examples of application aim to provide both mechanisms and motivation for researchers, particularly livestock researchers, to incorporate kinome analysis into their research programs.

## Introduction

Human and animal health research have each been revolutionized by technologies that enable global perspectives on cell biology. Omic approaches, an example of such technologies, conducted at a variety of biological levels have opened new frontiers for understanding biology as well as for diagnosis and treatment of disease. Ideally, the same omic approaches within human and animal health research fields can be applied with minimal barriers to translation allowing researchers to benefit from the advances made within each realm. This is true, to varying degrees, for the different omic disciplines. The effort required for the successful translation of the omic technologies does differ depending on characteristics of the biomolecule under consideration. That is to say, some omic technologies are more amenable to translation across species.

The opportunities enabled by broadly applicable omic technologies are particularly evident within nucleic acid-based investigations: DNA for genomics and RNA for transcriptomics. These approaches are based on research platforms that are largely species-independent such that identical omic technologies can be applied to virtually any organism. For example, the same basic approaches can be applied to either sequence the genome or define the transcriptome largely independent of the specific species. With that, technological advances within nucleic acid-based omics have great potential to offer immediate benefit to livestock researchers. For example, by serving as a catalyst for development of higher-throughput sequencing technologies, the Human Genome Project enabled determination of the genomes of livestock species including cattle (1), pigs (2), chickens (3), and turkeys (4). Likewise, within transcriptomics, there is a similar pattern of the development of technologies for traditional species of research priority (human and mouse) which are subsequently adopted by livestock researchers. This includes the use of transcriptional arrays (5), RNA-Seq (6), and single-cell RNA sequencing (7) to define transcriptional responses in livestock species.

The translation of technologies from human to animal health applications is of mutual benefit in that livestock researchers are empowered with cutting-edge technologies to advance their fields of study and the emerging data adds value and dimension to the human data by enabling species-comparative perspectives for human models of physiology and disease. Furthermore, as large animal models are representing an essential foundation for our understanding of human health and disease it is imperative that these species are investigated using advanced technologies (8). However, not all omic approaches share the same technological versatility for application across species, nor is there always the same level of motivation for their translation.

Within human health research, the priority for investigations of global cellular kinase (kinome) activity has been heavily motivated by the fact that kinases are intimately associated with many diseases and represent excellent drug targets (9). The “druggability” of kinases reflects both structural features of this class of enzymes that enables design of inhibitors as well as the central role of kinases as regulators of cellular responses and phenotypes (10, 11). In humans, many small-molecule protein kinase inhibitors have been approved or are advancing through clinical trials for the treatment of a diverse array of diseases (12). However, in animals the use of kinase inhibitor treatment has been limited. Select tyrosine kinase inhibitors have been approved for the treatment of cancer in companion animals (13, 14) as well as preliminary investigations of similar applications in horses (15). A category of kinase inhibitors, referred to as bumped kinase inhibitors (BKIs), target calcium-dependent protein kinases belonging to parasites of human and veterinary importance, including *Toxoplasma gondii*, *Plasmodium falciparum*, and *Cryptosporidium parvum* (16). BKIs have shown promising results as anti-parasitic drugs within food-animal species, including cattle (17) and pigs (18). From a safety perspective, BKIs represent the most likely usage of kinase inhibitors in livestock as the BKIs target non-mammalian kinases. Overall, however, the cost of these treatments relative to the value of the animals, as well as safety considerations (real, perceived, and regulatory) of such treatments, has prevented the use of kinase inhibitors as therapeutics in livestock animals.

The opportunities to employ kinase inhibitors as therapeutics is not, however, the sole benefit of kinome profiling. Kinome analysis also offers the unique advantage to understand the molecular basis of complex phenotypes. In part, this reflects the fact that kinase-mediated phosphorylation events succeed the transcriptional and post-transcriptional regulatory events that complicate the extraction of meaningful biological data from genomic and transcriptomic approaches. As kinase-mediated phosphorylation events often initiate cellular responses and phenotypes, defining host responses at the level of the kinome provides an opportunity for an unobstructed perspective of cellular events that anticipate, and are responsible for, organismal phenotypes. These same features also position kinases to serve as biomarkers of important phenotypes. Therefore, in spite of the somewhat restricted potential to the use of kinase inhibitor therapeutics in livestock, the other benefits of kinome analysis warrant effort to address the technological barriers that restrict the application of these approaches to livestock.

## Experimental Approaches to Define Kinase-Mediated Protein Phosphorylation

There are two primary methodologies that are employed to define kinase-mediated protein phosphorylation: phosphoproteome analysis, which characterizes the targets of the kinases, and kinome analysis, which quantifies the activities of the kinases. The different philosophical and technological basis of these approaches have been reviewed elsewhere (19). Each approach is associated with unique challenges and opportunities for application to livestock species (20).

### Phosphoproteome Analysis

Phosphoproteome investigations typically employ mass spectrometry to determine the phosphorylation status of proteins based on changes in molecular mass corresponding to the addition of a phosphoryl group (21). These types of phosphoproteomic characterizations can be performed in a largely species-independent manner as the basis for mass spectrometry analysis reflects changes to peptide characteristics (independent of their biological source) and that detailed predicted proteomes and their proteolytic peptide libraries are readily available for most species. Indeed, phosphoproteome characterizations have been applied to livestock to explore biological questions such as host-pathogen interactions (22), meat quality (23) and regulation of metabolism (24). The major technical limitations are the prohibitive costs and requirement for specialized equipment and personnel. The primary biological limitations are the challenges of defining dynamic patterns of phosphorylation within low abundance proteins, in particular those that reflect relatively small changes in the extent of phosphorylation of these proteins, a situation that often occurs within the context of signal transduction.

The phosphoproteome can be interrogated using antibodies that exclusively react with phosphorylated amino acids (i.e., serine, threonine, and tyrosine) or more specifically investigated using site-specific antibodies that only react with the protein in its phosphorylated state. This offers advantages of more quantitative assessment of priority phosphorylation events but it is ultimately limited by the availability and specificity of the antibody reagents. The availability of phosphorylation-specific antibodies is particularly problematic for livestock. While some commercially available phosphorylation-specific antibodies include information on reactivity across a range of species, some of which include livestock, this information is often unavailable or, in our experience, unreliable. Secondary challenges to this approach include technological obstacles to applying the antibodies in a high-throughput fashion; this is particularly challenging when using site-specific phosphorylation antibodies.

### Kinome Analysis

In contrast to phosphoproteome approaches, kinome analysis capitalizes on the fact that post-translational modifications represent enzymatic reactions. By providing an appropriate substrate, it is possible to quantify the activity of a particular kinase within the context of an enzymatic assay. As the specificity of many kinases is determined by the residues adjacent to the phosphorylation site (within 4 amino acid residues) (25, 26) it is theoretically possible to use short peptides as surrogate substrates for kinases. As short peptides are easily synthesized, relatively inexpensive and amenable to presentation in array formats this offers tremendous potential to develop peptide arrays that enable high-throughput analysis of cellular kinase activity. Early applications of kinase peptide arrays were performed to define phosphorylation sites and target-site specificity based on the ability of the kinases to modify peptides with shared sequence similarity. Once the utility and specificity of the arrays was established, these investigations evolved into applications to define cellular signaling responses within cellular lysates using kinome arrays. In this regard, the first true global kinome profiling experiment with peptide arrays defined signaling responses of human peripheral blood mononuclear cells (PBMCs) following stimulation of the innate immune receptor Toll-like receptor (TLR) 4 with its ligand lipopolysaccharide (LPS) (27); a major cell-wall constituent of Gram-negative bacteria. Notably, this pioneering investigation interpreted the emerging data from the perspective that each peptide represented a specific phosphorylation event of a particular protein rather than each peptide representing a general substrate for a specific kinase. That is to say, the data was interpreted from a phosphoproteome, rather than a kinome, perspective.

One of the considerable advantages of peptide arrays is that they are readily customized to represent the phosphorylation events that are of highest priority to the individual researcher. Designing the peptide arrays consists of selecting an appropriate number of phosphorylation sites from public phosphoproteome databases, such as PhosphoSite (28) and Phospho ELM (29). Within these databases, the phosphorylation events are typically presented as sequences of fifteen amino acids in length with the phosphoacceptor site in the central position. This format matches the design for most peptide arrays such that the information from these databases can be rapidly translated into a customized array.

One of the major hurdles with kinome analysis of livestock was that the commercially available arrays represented sequences derived from the human or mouse proteome and that information available within the phosphorylation databases was heavily biased toward those same species. The desire to perform kinome analysis of livestock, coupled with the scarcity of available experimental phosphoproteome information for these species, motivated alternate approaches for peptide array design. In particular, observation of the extent of conservation of the regions immediately surrounding phosphoacceptor sites indicated the potential to apply bioinformatic approaches to predict the phosphoproteome of species of interest.

## Platform Technologies for Generating Species-Specific Kinome Arrays

### Species-Specific Peptide Arrays

**FIGURE 1:**
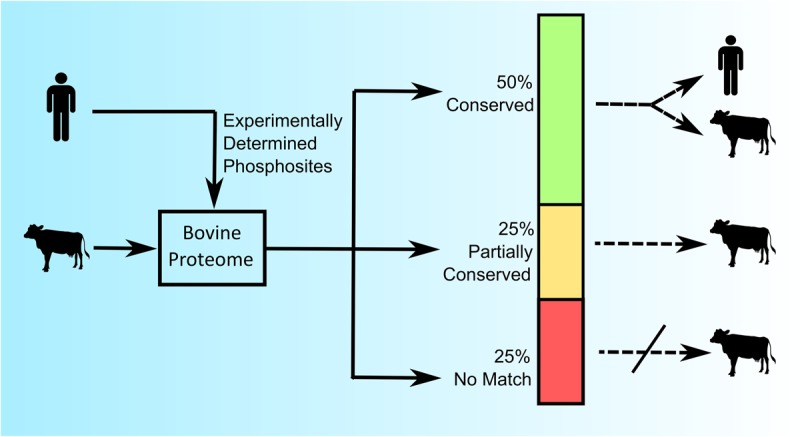
Comparative analysis of conserved and species-specific phosphorylation sites in the human and bovine proteome. Nearly 1,000 experimentally determined human phosphorylation sites (15 amino acids in length) from PhosphoSite were queried against the *Bos taurus* proteome using BLASTp (version 2.2.13) to identify protein homologs. “Conserved” represents 100% amino acid sequence identity; “Partially Conserved” represents less than 3 mismatches in the amino acid sequence; and “No Match” represents no identified homolog in the bovine proteome (30).

As a first exploration of the extent of conservation of phosphorylation sites across species nearly one thousand experimentally determined human phosphorylation sites (represented by a sequence of 15 amino acids with a centered phosphoacceptor site) were investigated within the bovine proteome (30). Of these phosphorylation sites, half were perfectly conserved across these two species (the same sequence within the context of a homologous protein), a quarter showed minor (less than three amino acids) sequence differences, and the final quarter had no identifiable protein homologs within the bovine proteome (Figure 1) (30). These results demonstrated both the potential, as well as the need, to construct species-specific peptides arrays.

The relatively high degree of conservation of phosphorylation sites across these species encouraged the potential to create species-specific peptide arrays. As such, in this example, it is possible to generate a bovine-specific peptide array by simply selecting phosphorylation sites whose peptide sequences are either absolutely conserved, or can be accommodated through minor species-dependent sequence adjustments. For example, a researcher could opt to limit the array to peptides whose sequences were absolutely conserved across the two species. In the described example, absolute conservation of sequence would represent half of the experimentally defined human phosphorylation sites as possible candidates for inclusion on the bovine peptide array (Figure 1). Notably, selecting phosphorylation sites whose surrounding sequences are absolutely conserved across the two species results in a tool that is equally appropriate for either humans or cattle use. Such “dual-species” arrays provide a common tool that facilitates direct comparison of results from each species and are therefore of particular value for species-comparative investigations. For example, the use of a pig/human dual-species peptide array demonstrated the ability to directly compare datasets from both human and porcine samples (31).

The list of potential peptides for a customized array can also be expanded through the inclusion, with appropriate modification, of peptides with minor species-specific sequence variations. In the previous example, the inclusion of the sequence-corrected peptides of this category would expand the list of peptides to include another quarter of the initial library of experimentally determined phosphorylation events but would also limit the application of that array to cattle (Figure 1).

The half of the peptides which were a perfect match, and the quarter which can be easily adopted through minor species-specific sequence variations, highlight the potential to create species-specific peptides arrays. The remaining quarter of human phosphorylation events which had no counterpart within the bovine proteome, speak to the need to create customized arrays for specific species. Consider, for example, if one were to utilize a peptide array representing human phosphorylation events to define kinome responses in bovine samples. In a best-case scenario the fraction of peptides (approximately 25%) representing human phosphorylation sites for which there is no functional equivalent within the bovine proteome would simply not be recognized by bovine kinases. While this would limit the efficiency of the array, there would be minimal consequences to the overall quality of the emerging data. Peptide arrays are typically applied to investigate relative differences in phosphorylation under different conditions rather than absolute levels of phosphorylation in a single condition; an unmodified peptide would appear as having no response to the stimulus under investigation. It would be more problematic if peptides representing phosphorylation events that do not exist within the bovine proteome were recognized and modified by bovine kinases, as this would imply the occurrence of phosphorylation events which, in reality, have no biological significance.

A recent investigation into the patterns of conservation of kinases and phosphorylation sites indicated there was greater evolutionary stability within the kinases as opposed to their phosphorylation sites (32). That is to say, a relatively stable infrastructure of kinases serves to modify a more malleable proteome. This would seem to support the potential for the presence of kinases with the ability to modify peptides that represent phosphorylation sites absent from within the proteome of that species. For these reasons, it is not recommended to utilize peptide arrays designed for a particular species to define the kinome of another species.

### Design Array for Phosphorylation Experiment (DAPPLE)

**FIGURE 2:**
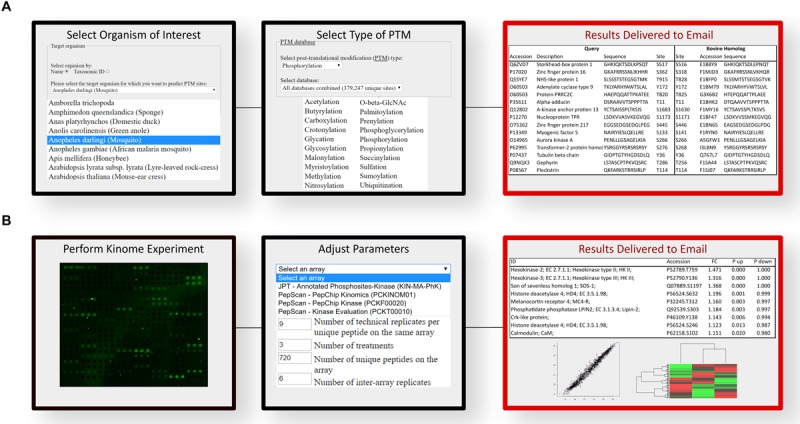
Computational workflow for the prediction of phosphopeptides and analysis of kinome array data. **(A)** DAPPLE2 facilitates the prediction of post-translational modification sites in proteins. Specific to kinome technology, DAPPLE2 can predict and generate a list of all putative phosphopeptides from the selected species of interest. **(B)** PIIKA2 software was custom designed for the transformation, deconvolution, statistical analysis and visualization of kinome array data. PIIKA2 results returned to the user include various statistical analyses and data visualization such as principal component analysis plots, scatter plots, and hierarchical clustering heat maps. Both software platforms are freely accessible to all researchers at http://saphire.usask.ca/saphire/.

A software platform called Design Array for Phosphorylation Experiment (DAPPLE) was created to streamline and automate the process of developing species-specific peptide arrays. DAPPLE utilizes BLAST to analyze the sequence similarity between experimentally determined phosphorylation sites in other organisms against the proteome of the species of interest (33). DAPPLE returns to the user a list of peptides containing a putative phosphoacceptor site for inclusion on a species-specific array. This peptide library typically ranges in size from thousands to tens of thousands. DAPPLE was later expanded into DAPPLE2 (34), to improve on the original program by surveying a greater number of phosphorylation site datasets as well as enabling consideration of other forms of post-translational modification. As current arrays typically contain approximately a thousand unique peptides, it can be a daunting task to manually select these from the tens of thousands that are typically present within the DAPPLE output files. DAPPLE2 facilitates this process by providing the user with gene ontology terms, signaling pathways, and indicators of the confidence of the predicted phosphorylation site including sequence identity within the phosphorylation region and the homology of the protein in which the phosphorylation site is contained. DAPPLE2 also provides information on the number and nature of supporting publications for the phosphorylation event. The nature of the supporting papers being further defined on the basis of whether the source publication represents high- or low-throughput approaches. Greater confidence and priority are assigned to phosphorylation events characterized using low-throughput approaches (like site-directed mutagenesis) as opposed to high-throughput global characterizations of the phosphoproteome. This information also allows researchers to simplify peptide selection based on clear and rational criteria relating to both the biological function and confidence in the predicted phosphorylation site. For example, a user could specify the selection of phosphorylation events which are involved in metabolism, which are absolutely conserved within the target species, and are supported by at least three publications, one of which representing a low-throughput study. Based on the number of peptides meeting these criteria, the user can choose to alter the selection criteria until a suitable number of peptides are identified. Using this approach, it is possible for a biologist with minimal background in bioinformatics to design a species-customized array, with or without an emphasis on specific biological processes, in a matter of hours. The workflow interface of DAPPLE2 is illustrated (Figure 2A).

It is also worthy to note that peptide arrays designed on the basis of predicted phosphoproteomes are inherently less reliable than those reflecting phosphorylation sites characterized through low-throughput experimental approaches. The conservation of a matching sequence, in a homologous protein, as an experimentally determined phosphorylation site is not absolute assurance that the same phosphorylation, and by extension the same biological outcome, will occur in the target species. With that appreciation, our central philosophy is that kinome analysis is a tool, similar to other omic technologies, employed to generate data that lead to novel hypotheses that are then further substantiated by independent approaches.

### Platform for Integrated, Intelligent Kinome Analysis (PIIKA)

Interpreting the results of high-throughput analyses that involves high-level statistics on thousands of data points can be extremely difficult. As such, extraction of meaningful biological information is a significant challenge to any omic approach, with kinome analysis being no exception. To this end, a software tool, Platform for Intelligent, Integrated, Kinome Analysis (PIIKA), and our latest version PIIKA2, was created with specific consideration of the technical and biological characteristics of kinome peptide arrays (35). An easy to use web-based interface allows biologists lacking a strong background in data science to upload kinome datasets to perform various analyses and tests: data normalization, evaluation of how well different experimental groups cluster together, identification of peptides with consistent phosphorylation patterns amongst experimental groups, view false negative probabilities, positive and negative predictive values for t-tests between pairs of samples, and readily quantify experimental reproducibility (36). PIIKA2 includes various statistical analyses such as fold-change analysis, principal component analysis, determination of Euclidean distance between groups, and hierarchical clustering (35). Visualization tools within PIIKA2, such as volcano plots, scatterplots and heat maps aid in the selection process and statistical interpretation as well as being readily presentable and easy to understand, in comparison to the raw output. The output files of PIIKA2 are compatible with software platforms for higher-level analysis, such as pathway analysis. The workflow interface of PIIKA2 is illustrated (Figure 2B).

## Applications of Kinome Analysis

**FIGURE 3:**
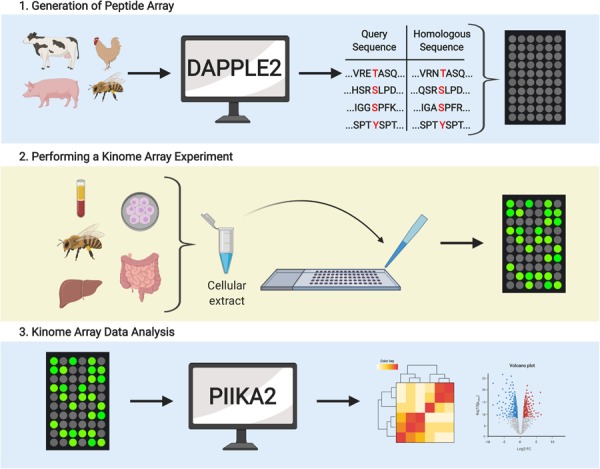
Overview of the kinome array workflow. Schematic representation of the three main components of the kinome array pipeline. **(1)** Computational workflow (DAPPLE2) for predicting putative phosphoacceptor peptides for spot printing on peptide microarrays. **(2)** Representation of the various cells, tissues and whole organisms in which cellular extracts can be prepared to quantify kinase-mediated phosphorylation using peptide arrays. **(3)** Computational workflow (PIIKA2) for transforming, statistical analysis and graphical visualization of kinome array data.

In the decade following the development of the species-specific peptide arrays there has been a wealth of publications that highlight the utility of kinome analysis of livestock species (Table 1). These investigations explore a variety of species, biological questions, and sample matrices. In the subsequent sections we present examples to highlight the diversity within each of these. Species-specific peptide arrays have been created and applied for the primary food-associated livestock animals (i.e., cattle, pigs, and chickens). In terms of biological questions, the application of kinome analysis to livestock has focused on two issues of greatest significance to the industry, infectious diseases, and response of animals to stresses associated with modern management practices. Within these investigations, a variety of biological samples have been considered which range in complexity from immortalized cell lines and highly purified primary cells to complex cell populations like PBMCs and tissue samples (i.e., muscle and intestine), and even whole organism kinome profiling. These examples collectively highlight the utility and robustness of the technology. The workflow for the design, application, and interpretation of peptide arrays for kinome analysis is illustrated (Figure 3).

**TABLE 1:** Applications of peptide array kinome analysis to livestock species.

		**Sample**			
**Species**	**Challenge**	**Cell culture**	**PBMCs**	**Muscle**	**Intestine**
Bovine (cattle)	Infectious disease	(57, 58, 61, 109)	(74)		(70)
	Stress		(90)		
Swine (pig)	Infectious disease				(93)
	Stress				
	Other		(31)		
Avian (chicken)	Infectious disease	(64, 66, 110, 111)		(75)	(76–78, 112, 113)
	Stress			(92)	
	Other				(103, 114)
Ovine (sheep)	Infectious disease	(94, 115)			(94)

### Kinome Analysis of Infectious Disease

As the activation of innate immune responses relies heavily on phosphorylation-mediated signal transduction, kinome analysis is a particularly appropriate approach for defining host responses to microbial pathogens (37). Given the importance of kinase-mediated signaling in the activation of immune responses, it is not surprising that many pathogens, in particular those that result in chronic infections, can subvert protective host immune responses using their own effector kinases, and phosphatases in addition to utilizing other virulence factors that function to manipulate host signaling either directly or indirectly (38–40). These tactics can represent a critical obstacle in the development of effective vaccines and/or immunotherapeutics. These limitations are potentially addressed through a more detailed understanding of the host-pathogen interaction; understanding the molecular mechanisms of these interactions can guide rationale development of vaccines and/or therapeutics (particularly, in the form of kinase inhibitors), as well as facilitating the identification of biomarkers that anticipate the susceptibility, resistance, severity, or outcome of infection. With this, it is not surprising that early examples of kinome analysis through peptide arrays were performed in the context of investigating the host-pathogen interaction.

## Kinome Analysis of *Ex Vivo* Infection Models

To enable greater opportunity for insight into host immune responses to pathogenic challenge, the earliest kinome investigations of host-pathogen interactions were often conducted within simplified infection models, like cell lines or highly purified primary cell populations. The biological significance of the findings of these investigations were then typically validated within *in vivo* infection models through either direct confirmation of biological responses or through the effective use of therapeutics informed by the *ex vivo* investigation.

### *Mycobacterium avium* subsp. *paratuberculosis* (*ex vivo*)

Johne’s disease, a chronic inflammatory disorder of the small intestine of ruminants, is caused by *Mycobacterium avium* subsp. *paratuberculosis* (41). *M. paratuberculosis* is an intracellular pathogen that achieves chronic infection through subversion of the host immune response (42, 43). In host macrophages, *M. paratuberculosis* inhibits phagosome maturation (44) to promote its intracellular survival and alters cellular signaling to inhibit the normal bactericidal activity of the host cell (45). Inhibiting interferon gamma (IFNγ) expression and signaling is of central importance to intracellular pathogens, including *M. paratuberculosis*, to evade cell-mediated immunity (43, 46). A number of pathogens including *Trypanosoma cruzi* (47), *Leishmania donovani* (48), and *Mycobacterium avium* (49) block IFNγ responsiveness by dampening the expression of the IFNγ receptor. Induced expression of suppressor of cytokine signaling (SOCS), a key regulator in the IFNγ signaling pathway, has also been observed following infection with various pathogens including *Toxoplasma gondii* (50), *Burkholderia pseudomallei* (51), and Group A *Streptococcus* (52). IFNγ treatment of macrophages prior to *M. paratuberculosis* infection promotes their ability to clear infection, but the same treatment is ineffective post-infection (53, 54). This suggests that *M. paratuberculosis* infection desensitizes infected cells to IFNγ stimulation. Highly analogous to the situation with IFNγ, prophylactic stimulation of TLRs on macrophages prior to infection enhanced bactericidal activity against *M. tuberculosis*, but was ineffective post-infection (55); *in vivo*, *M. paratuberculosis* infected sheep show differential expression of TLRs suggesting this pathogen also targets these innate immune pathogen-recognition receptors to evade protective host responses (56). Among the earliest applications of the species-specific peptide arrays were two investigations to determine the extent and mechanisms by which *M. paratuberculosis* influences the responsiveness of bovine macrophages to both endogenous and exogenous activators of the innate immune response: IFNγ (57) and CpG-ODN (a TLR9 agonist) (58), respectively. Both investigations employed an infection model of primary bovine monocytes that enabled a homogeneous and biologically relevant cell population.

**FIGURE 4:**
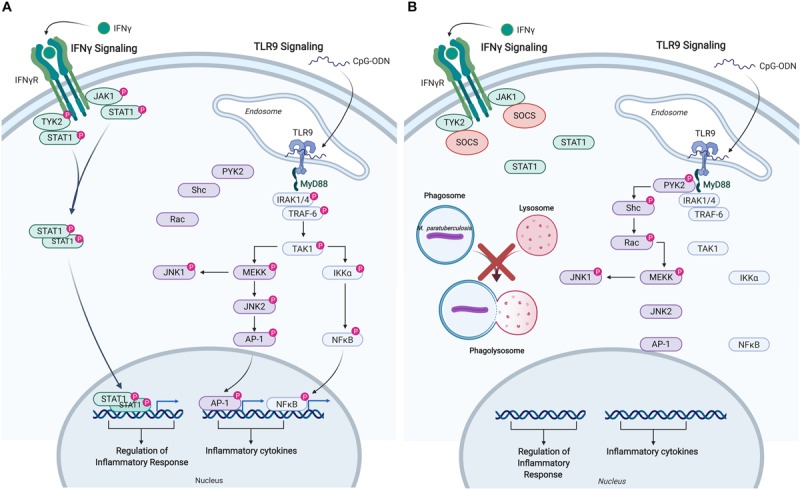
Immune signaling pathways in bovine monocytes. Schematic representation of the IFNγ and TLR9 signaling pathways in response to exogenous stimuli and *M. avium* subsp. *paratuberculosis* infection. **(A)** Signaling pathways activated in uninfected bovine monocyte following stimulation with IFNγ, and TLR9 agonist CpG-ODN. Both pathways lead to the downstream phosphorylation of unique intermediates terminating with the translocation of distinct transcriptional activators into the nucleus to induce the expression of pro-inflammatory responses and cytokines. **(B)** *M. paratuberculosis* infection of bovine monocytes interferes with IFNγ signaling near the receptor by inducing the expression of SOCS, which disrupts JAK-STAT signaling. *M. paratuberculosis* infection also dysregulates TLR9 signaling by shunting the response toward the PYK2 pathway effectively blocking the induction of pro-inflammatory responses via MYD88. “P” designates protein in its phosphorylated state. Figure is adapted from Arsenault et al. (57, 58) and generated using BioRender. AP-1, adaptor protein complex 1; CpG-ODN, cytosine triphosphate-guanine triphosphate oligodeoxynucleotide; IFN, interferon; IKKα, inhibitor of nuclear factor kappa-B kinase subunit alpha; IRAK, interleukin-1 receptor-associated kinase 1; JAK, janus kinase; JNK, c-Jun N-terminal kinase; MEKK, MAPK/ERK kinase; MYD88, myeloid differentiation primary response protein; NFκB, nuclear factor kappa B; PYK, protein-tyrosine kinase; RAC, Ras-related C3 botulinum toxin substrate; SHC, Src homology 2 domain-containing-transforming protein; SOCS, suppressor of cytokine signaling; STAT, signal transducer and activator of transcription; TAK, TGF-beta-activated kinase; TRAF, TNF receptor-associated factor; TYK, non-receptor tyrosine protein kinase; TLR, Toll-like receptor.

The responsiveness of uninfected and *M. paratuberculosis* infected monocytes was measured by induction of released cytokines: TNFα in response to IFNγ, and IL-10 in response to CpG-ODN. IFNγ stimulation of uninfected monocytes caused a dramatic release of TNFα while CpG-ODN stimulation induced the release of IL-10. By contrast, *M. paratuberculosis* infection of monocytes significantly diminished responsiveness to IFNγ and CpG-ODN. Kinome profiling of the uninfected monocytes indicated activation of signaling pathways classically associated with each ligand; JAK-STAT signaling in response to IFNγ and TLR signaling in response to CpG-ODN (Table 2) (57, 58). Infected monocytes, however, failed to induce JAK-STAT signaling responses indicating that *M. paratuberculosis* blocks IFNγ responsiveness at, or near, the IFNγ receptor (Figure 4) (57), and CpG-ODN induced signaling was redirected away from traditional TLR pathway into Pyk2-mediated signaling (Table 2). Further investigation revealed that as early as 1-h post-infection SOCS1 and SOCS3 expression significantly increased with subsequent decreased expression of the IFNγ receptor by 18 h post-infection. These data suggest that each of these events desensitized *M. paratuberculosis*-infected cells to IFNγ stimulation. In contrast to the complete repression of IFNγ-induced signaling, *M. paratuberculosis* infection redirected CpG-ODN signaling to an early intermediate of TLR signaling, Pyk2 (59). This redirection was confirmed through phosphorylation-specific antibodies as well as functional assays (58). As Pyk2 signaling had not been previously implicated in *M. paratuberculosis* infection this highlights the power of kinome technology for novel discovery.

**TABLE 2:** Bovine monocytes differentially respond to IFNγ and CpG-ODN depending on *M. avium* subsp. *paratuberculosis* infection status.

		**Uninfected monocytes**				***M. paratuberculosis*- infected monocytes**			
		**Up**		**Down**		**Up**		**Down**	
	**↕**	**#**	***p* value**	**#**	***p* value**	**#**	***p* value**	**#**	***p* value**
**IFNγ stimulation**									
JAK-STAT signaling pathway	16	15	0.002	1	1	4	0.8	7	0.3
Gene expression of SOCS	6	6	0.03	0	1	1	0.9	3	0.3
**CpG-ODN stimulation**									
TLR signaling	18	14	0.02	4	1	2	1	8	0.06
Pyk2 signaling	10	3	1.0	7	0.07	9	0.01	1	1

*Pathway analysis was completed using InnateDB (116). Functional enrichment and pathway prediction (*p* value) is based on the number of differentially phosphorylated proteins from the experimental dataset represented within the annotated pathway in the database. Reported in the table are the total number of differentially phosphorylated peptides (↕), when compared to uninfected control cells, belonging to each pathway including the total number of peptides (#) within that pathway that show increased (Up) and decreased (Down) phosphorylation (58).*

### *Mycoplasma bovis* (*ex vivo*)

*Mycoplasma bovis* (*M. bovis*) is responsible for a number of diseases of cattle including pneumonia, mastitis, arthritis, and abortion (60). *M. bovis* typically functions as a respiratory pathogen entering the host through lung epithelial cells and subsequently establishing residence within blood monocytes. Persistence of *M. bovis* within the monocytes affords the opportunity for protected dissemination throughout the host.

The mechanisms by which *M. bovis* establishes persistent infection of host immune cells had yet to be fully described. Given the success of kinome analysis in determining the mechanisms by which *M. paratuberculosis* achieves persistent infection of bovine monocytes, a similar *ex vivo* investigation was performed for *M. bovis*. One of the key findings of the kinome analysis was the implication by the kinome data that *M. bovis* sought to influence apoptosis through manipulation of the caspase system. Specifically, the signaling events induced by *M. bovis* were consistent with an anti-apoptotic outcome. Functional assays of both spontaneous and induced apoptosis confirmed the kinome results in that *M. bovis*-infected cells had decreased rates of spontaneous apoptosis as well as lower levels of induced apoptosis in response to pro-apoptotic stimuli (61). The influence of *M. bovis* on apoptosis was suggested as a mechanism to prolong bacterial survival as well as to enable dissemination of the pathogen throughout the host.

### *Salmonella* (*ex vivo*)

Poultry is the most significant contributor to food-borne Salmonellosis in humans (62). Colonization of chickens with Salmonella results in a rapid (less than 4 h) inflammatory response that evolves into an asymptomatic, persistent infection during which time the bacterium is continuously shed in feces (63). This underscores the capacity of Salmonella to rapidly evade host innate immune defenses and persistently colonize the avian host without eliciting an active immune response. Understanding this host-pathogen interaction is essential for developing novel intervention strategies to eradicate infection especially as antibiotic-resistance and the restricted use of antibiotics in the poultry industry both continue to grow.

**FIGURE 5:**
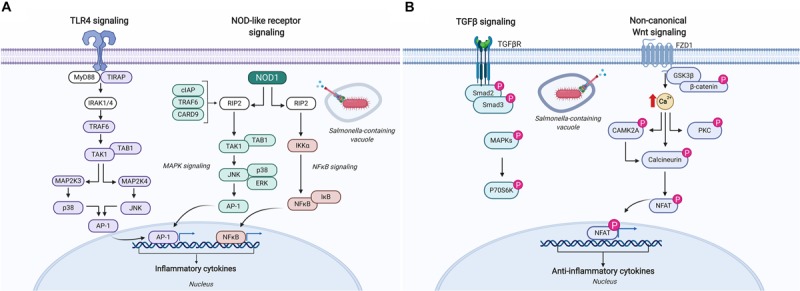
Schematic representation of the immune signaling responses occurring in chickens infected with Salmonella. **(A)** Salmonella infection of chicken macrophages *in vitro* activates TLR4 and NOD-like receptor signaling pathways leading to the activation of unique transcriptional activators involved in inducing pro-inflammatory responses (64). **(B)** Kinome analysis of cecal tissue from Salmonella-infected chickens revealed differential phosphorylation of TGF-β and non-canonical Wnt signaling pathways suggesting a dampening of pro-inflammatory responses that supports Salmonella evading host immune defenses and establishing a persistent local infection (76). Figure is adapted from He et al. (64) and Kogut et al. (76); additional signaling responses have been characterized through biochemical analyses of TLR4, CARD9, and the NLRP3 inflammasome in bone marrow-derived macrophages and are described elsewhere (117–119). Protein identities in the figure that are colored (i.e., purple, blue, and pink) were identified as differentially phosphorylated (*p* < 0.05) (64, 76). Figure is adapted from KEGG Pathway Mapper (https://www.genome.jp/kegg/mapper.html), and generated using BioRender. “P” designates protein in its phosphorylated state. AP-1, adaptor protein complex 1; CAMK2A, calcium/calmodulin-dependent protein kinase type II subunit alpha; CARD, caspase recruitment domain-containing protein; cIAP, cellular inhibitor of apoptosis; ERK, extracellular signal-regulated kinase; FZD1, Frizzled-1; GSK3β, glycogen synthase kinase-3 beta; IκB, NF-κB inhibitor alpha; IKKα, inhibitor of nuclear factor kappa-B kinase subunit alpha; IRAK, interleukin-1 receptor-associated kinase; JNK, c-Jun N-terminal kinase; MAP, mitogen-activated protein kinase; MYD88, myeloid differentiation primary response protein; NFAT, nuclear factor of activated T-cells; NFκB, nuclear factor kappa B; NOD, nucleotide-binding oligomerization domain-containing protein; P70S6K, ribosomal protein S6 kinase beta-1; PKC, protein kinase C alpha type; RIP, receptor-interacting serine/threonine-protein kinase; Smad, mothers against decapentaplegic homolog; TAB, TAK1-binding protein; TAK, mitogen-activated protein kinase; TGFβ, transforming growth factor beta; TGFβR, TGFβ receptor; TIRAP, toll/interleukin-1 receptor domain-containing adaptor protein; TRAF, TNF receptor-associated factor.

Kinome analysis has provided substantial contributions in understanding how Salmonella evades innate immune defenses and perturbs host cell signaling *in vitro* to gain the advantage. In one particular study, chicken macrophages were infected with *Salmonella* Enteritidis and *S.* Heidelberg for 1.5, 3, and 7 h to identify species-specific host responses (64). Kinome analysis indicated that phosphorylation events associated with lysosome and phagosome processes were significantly different between these two serovars, specifically suggesting that *S.* Enteritidis more effectively alters these signaling pathways to evade host innate defenses. This finding is consistent with the greater intracellular survival of *S.* Enteritidis in chicken macrophages *in vitro* compared to *S.* Heidelberg (65). Pathway analysis of the differentially phosphorylated peptides in Salmonella infected macrophages also identified a number of common pathways upregulated by both serovars that are potentially involved in pathogen response and control including: increased dephosphorylation (i.e., activation) of inducible nitric oxide; activation of TLR4 and TLR5 pathways including many of the adaptor and intermediate signaling proteins involved in the signal cascade leading to NF-κB activation (Figure 5); and reduced phosphorylation (i.e., activation) of NLRP3 – a major hub in the inflammasome. Salmonella evasion of innate responses in macrophages was most evident in the dephosphorylation (i.e., inactivation) of the adaptor protein caspase recruitment domain-containing protein 9 involved in mediating NOD-like receptor signaling leading to activation of cell apoptosis and the production of NF-κB, and the continued perturbation of mitogen-activated protein (MAP) kinases at all time points (Figure 5A). These findings provide the basis for developing and testing novel therapeutics that target these pathways and gene products to potentially boost innate immune defenses that restrict Salmonella intracellular persistence.

A follow up *in vitro* study by the same authors focused their analysis on phosphorylation targets associated with proteins in the calcium/calmodulin signaling pathway (66). Both *S.* Enteritidis and *S*. Heidelberg infection of chicken macrophages resulted in differential phosphorylation of peptides associated with calcium/calmodulin signaling pathway suggesting Salmonella dysregulates this pathway to promote its intracellular survival. This conclusion was further supported by the observation that treatment of chicken macrophages with a calmodulin inhibitor both inhibited nitric oxide production and promoted the intracellular survival of Salmonella. Taken together, these *in vitro* studies illustrate how kinome technology can be applied to investigate global changes in host cell signaling pathways to specifically identify mechanisms exploited by pathogens to evade host innate immune defenses, and those in which the host activates in an attempt to control infection.

## Kinome Analysis of *In Vivo* Infection Models

While the early kinome investigations of host-pathogen interactions prioritized *ex vivo* infection models, there was early evidence of the opportunity for consideration of samples of greater biological complexity. Specifically, that the pioneering investigation of peptide arrays described signaling responses within PBMCs (27). Encouraged by the results of the kinome analysis within *ex vivo* models, and emboldened by the results of the characterization in PBMCs, the technology was translated to *in vivo* infection models. The following examples represent kinome analysis of different biological matrices from *in vivo* infection models: PBMCs, intestinal biopsies, and muscle samples. Additionally, in the unique situation of investigating host responses of an insect to pathogenic challenge, whole organism kinome profiling is also described.

### *Mycobacterium avium* subsp. *paratuberculosis* (Intestinal Samples)

The majority of cattle infected with *M. paratuberculosis* do not develop clinical Johne’s disease. This indicates the ability of animals to mount a local immune response that controls the infection. The specific mechanisms by which these animals resist infection are not clearly defined. Understanding the molecular basis of a protective response would provide valuable guidance in the efforts to develop vaccines and therapeutics. It was hypothesized that differences in this host-pathogen interaction in the early stages of infection at the local site of infection in the small intestine determine the nature and efficiency of the induced immune response. To study the host- *M. paratuberculosis* interaction at the local site of infection in the bovine host, a novel intestinal segment model was employed (67). This intestinal segment model enables targeted delivery of a defined dose of a pathogen contained to a specific region of the gut (68, 69). In this study, intestinal segments were surgically isolated in the ileum, the site of persistent *M. paratuberculosis* infection. Additionally, uninfected intestinal segments were prepared proximal to the *M. paratuberculosis*-infected segments of the same animal serving as valuable intra-animal, syngeneic controls.

Kinome analysis was performed on samples from the uninfected and *M. paratuberculosis*-infected segments at 1-month post-infection. The datasets emerging from kinome analysis on these ileal intestinal samples clustered into two distinct groups, indicative of the occurrence of distinct cellular responses to *M. paratuberculosis*. These differences in signaling corresponded to innate immune and interleukin (IL-1, IL-4, IL-6, and TGF-β) signaling pathways as well as differences in the Wnt/β-catenin pathway. The distinct signaling responses to *M. paratuberculosis* at the site of infection were also reflected at the level of the organismal *M. paratuberculosis*-specific immune responses where the experimental animals could be classified into two distinct groups based on distinct antibody, T cell proliferation, and IFNγ responses (70). Most significantly, the distinct patterns of cell signaling anticipated the differences in the *M. paratuberculosis*-specific immune responses. Understanding the cellular mechanisms that determine the balance between cell-mediated and antibody responses could be of considerable importance in the development of treatments for *M. paratuberculosis* as well as providing a novel method for rationale selection and/or design of mucosal vaccines and adjuvants.

In addition to the biological insight that was provided into *M. paratuberculosis* infection, this investigation represented a key step in the evolution of the application of kinome analysis for understanding host-pathogen interactions. By demonstrating that critical differences in signaling could be detected in response to stimuli of the intact animal motivated subsequent efforts to apply kinome to define responses occurring within the context of the intact host.

### Bovine Viral Diarrhea Virus (PBMCs)

As previously described, many pathogens, in particular those that result in persistent infections, utilize immunosuppression as a significant component of their pathogenic mechanism. Within this, a common theme is to limit the ability of the infected host to produce, or respond to, interferons. Bovine viral diarrhea virus (BVDV) is responsible for some of the most significant losses to the global cattle industry (71). While BVDV causes persistent infection of cattle, there is debate of the extent and mechanisms by which the pathogen impacts host immune responses.

Bovine viral diarrhea virus strains cluster into two genotypically distinct clades, BVDV1 and BVDV2, with further sub-division of each genotype into cytopathic (cp) and non-cytopathic (ncp) phenotypic biotypes on the basis of their lytic activity to tissue culture epithelial cells (72). In general, cpBVDV strains are associated with the activation of IFN responses while there is less consensus on whether this is also true for ncp-BVDV strains (72–74). It is also important to keep perspective that manipulating the induction of these cytokines is just one possible mechanism by which ncp-BVDV could manipulate this aspect of the host immune response, that blocking the ability of the infected cells to respond to these signals, as observed for *M. paratuberculosis* and other pathogens, is another option to functionally negate this host immune response.

An investigation was conducted to determine the occurrence and functionality of interferon responses following the challenge of cattle with ncp-BVDV. There were three aspects to this characterization: (1) defining levels of interferon in response to challenge with ncp-BVDV, (2) kinome analysis of PBMCs from infected calves to investigate interferon-associated signaling, and (3) transcriptional analysis of interferon-regulated genes at time points corresponding to the IFNγ and IFNα responsive phases of acute BVDV infection. This collectively covers the induction of interferon release, the ability of these cytokines to induce signaling events within immune cells, and the functional consequences of these signaling events.

In response to the infection of cattle with ncpBVDV2-1373 there were significant increases in serum levels of both IFNγ and IFNα. The functionality of these responses was dually supported at the levels of both signal transduction and gene expression; there was clear evidence for activation of classic IFN-activated signaling pathways, as well as induced expression of IFNγ and IFNα regulated genes, within the PBMCs of the infected animals relative to the age-matched controls (74). Dampening of the IFNγ responsiveness of peripheral blood immune cells had also been proposed as an element of the pathogenic mechanism of BVDV (72) but kinome analysis of PBMCs from BVDV-infected cattle indicated activation of IFNγ induced signaling which was further confirmed through induced-expression of IFNγ regulated genes (74).

This paper was highly significant in demonstrating the ability to monitor host responses to pathogens within a cell population that is readily available for repeated sampling in a non-lethal fashion. As will be discussed later, PBMCs seem to hold tremendous potential for kinome investigations to determine responses to a number of stimuli as well as for the identification of phosphorylation-associated biomarkers.

### Salmonella Infection of Chickens (Muscle Samples)

*Salmonella enterica* serovar Typhimurium (*Salmonella* Typhimurium) infection of young chickens results in asymptomatic colonization of the cecum accompanied by persistent fecal shedding. Despite the apparent disease-free state of these infected birds, it was hypothesized that the local colonization of the cecum has systemic effects influencing the physiology of the avian host. To address this, kinome analysis was completed on breast muscle collected from Salmonella challenged and uninfected broiler chickens to identify differentially phosphorylated peptides (75). Biological pathway analysis of the differentially phosphorylated peptides revealed that host metabolic pathways were significantly dysregulated in breast muscle during the early stages of infection (<3 weeks post-infection). Specifically, pathways associated with decreased energy currency (i.e., glucose metabolism, and intermediates shared between insulin and mTOR pathways), fatty acid metabolism (via AMPKα signaling) and immune-related pathways (i.e., Fc receptor and TLR signaling). Despite the apparent lack of clinical signs associated with Salmonella infection in these chickens, kinome analysis identified profound systemic effects of infection on skeletal muscle suggesting colonization negatively affects the physiology of the avian host with specific ramifications on meat quality.

A number of studies have used kinome technology to identify the cell signaling pathways exploited by Salmonella to support its persistence in the intestines of broiler chickens. In one particular study, cecal tissue collected from Salmonella-challenged broiler chickens revealed an up-regulation of the pro-inflammatory cytokine gene *IL-6* early in infection (48 h post-infection) that quickly regressed by 4 days post-infection as the anti-inflammatory cytokine gene *TGF-*β*4* was up-regulated and remained significantly higher at 7, 10, and 14 days post-infection (76). Kinome analysis was used to identify the signaling pathways and mechanisms responsible for this persistent local anti-inflammatory state. Cecal tissue extracts from Salmonella-infected and control chickens applied to chicken species-specific kinome arrays revealed significant kinase-mediated phosphorylation of peptides associated with canonical Wnt/β-catenin, non-canonical Wnt/Ca^2+^, and TGF-β signaling pathways at 4 days post-infection (Figure 5B). Closer investigation into individual phosphorylation events on the kinome array showed increased phosphorylation of nuclear factor of activated T cell (NFAT) peptides in addition to dephosphorylation of IKK and NF-κB suggesting Salmonella targets key host proteins to suppress the activation of pro-inflammatory cytokine responses thus promoting an anti-inflammatory microenvironment to support its persistence within this tissue (Figure 5B).

To further elucidate host immune signaling pathways elicited by the avian host following *Salmonella* Enteritidis infection, cecal tissue was collected from Salmonella challenged broiler chickens and extracts applied to chicken species-specific kinome peptide arrays (77). Differentially phosphorylated peptides at 4 days post-infection belonged predominantly to two immune-related pathways: T cell signaling and JAK-STAT pathways. Further characterization of these differential phosphorylated peptides led to the proposed mechanism whereby dephosphorylation of phospholipase c-G1 fails to activate either NF-κB or NFAT leading to the inhibition of local pro-inflammatory responses providing Salmonella with an immune privileged site to establish persistent infection. In addition to elucidating proposed mechanisms associated with immune evasion, kinome analysis has also helped identify host cell signaling responses associated with increased natural resistance to Salmonella infection. Intestinal tissue extracts from chickens categorized as high and low bacterial burden were applied to kinome peptide arrays and differentially phosphorylated peptides were comparatively analyzed. Pathway analysis revealed that intestinal tissue from chickens with low bacterial burden, when compared to high bacterial burden, up-regulated pathways associated chemokine signaling, FcεRI signaling, focal adhesion, insulin signaling, JAK-STAT signaling pathway, MAP kinase signaling, neurotrophin signaling, and T cell receptor signaling (78). These analyses suggest that early activation of these pathways at the local site of infection are associated with increased natural resistance to Salmonella infection in the avian host. Continued investigation and validation into these gene products and pathways will further advance our understanding of intracellular Salmonella infection in an effort to improve animal health and meat quality, and provide greater food safety to consumers.

### Varroa Mite Infestation of Honeybees

While not a traditional livestock species, honeybees are key contributors to food production with approximately a third of food crops depending on them for pollination. As such, there is considerable concern over the trend of worldwide declines in honeybee populations and health (79). Infestation by Varroa mites is typically regarded as the most detrimental threat to honeybee health. A current priority of the honeybee industry is to identify mechanisms and biomarkers of Varroa mite tolerance to inform breeding efforts toward this phenotype. To this end, a honeybee-specific peptide array was developed to enable investigation of kinome responses to Varroa mite challenge. The development of a peptide array for an insect represented a significant advancement in the prediction of phosphorylation sites in a species of interest. This was achieved through consideration of experimentally determined phosphorylation sites from a variety of species but perhaps most importantly, from *Drosophila melanogaster* which was the closest relative for which phosphorylation sites had been defined.

The application of this array to uninfested bees representing colonies of defined, differentially sensitivities to Varroa mite infestation revealed unique signaling profiles between bees of the two phenotypes (80). That is to say, the differences in phenotypes were reflected at the level of whole organism signaling profiles, supportive of the potential to use these differences as biomarkers to guide breeding efforts. Furthermore, bees of the different phenotypes demonstrated distinct signaling responses to Varroa mite challenge. Gene ontology analysis of the peptides which were differentially phosphorylated between the bees of the two phenotypes indicated that the distinct susceptibilities to Varroa mite infestation did not reflect compromised immunity within the uninfested Varroa mite susceptible bees. Instead, there was evidence that mite infestation results in immune suppression specifically within bees of the susceptible phenotype. This immunosuppression increases the susceptibility of these bees to secondary viral infections, including those carried by the Varroa mites. The demonstration of more diverse viral infections in mite-infested, susceptible adult bees would seem to support this hypothesis (80).

## Kinome Analysis for Identification of Antimicrobial Therapeutics

While many kinase inhibitors used in the context of cancer chemotherapies, there is emerging appreciation of the potential to repurpose licensed kinase inhibitors, including as antibiotics and antivirals (81). This includes, but is not limited to, pathogens that directly impact host signaling through the use of kinase effector molecules. In these instances, there is the opportunity to use kinase inhibitors designed for bacterial kinases as antimicrobials (82). As previously mentioned, the BKIs which are specific for parasitic targets are under active investigation for the treatment of a number of human and veterinary infections. It is also possible to use kinase inhibitors to impact host signaling to promote the clearance of a pathogen. For example, imatinib, an FDA approved chemotherapeutic kinase inhibitor, facilitates clearance of *Mycobacterium tuberculosis*, the causative agent of tuberculosis, from a human fibroblast cell line (83).

Kinome analysis of host-pathogen interactions also has the potential to identify signaling pathways impacted by infection which, when acted upon through kinase inhibitors, have the potential to promote more effective clearance of the pathogen. For example, kinome analysis of *M*. *paratuberculosis*-infected monocytes indicated that the pathogen redirects TLR signaling through the Pyk2 pathway, an action that would seem to serve the benefit of the pathogen. Based on this hypothesis, Pyk2 inhibitors were investigated as potential therapeutics of this chronic infection. Consistent with the hypothesis, treatment of *M*. *paratuberculosis*-infected cells with Pyk2 inhibitors promoted clearance of the pathogen (58).

In a pair of investigations of high consequence pathogens, kinome analysis was applied in an effort to identify therapeutic targets. Kinome analysis of human hepatocytes to Ebola infection identified VEGF signaling as a critical component of the pathogenic mechanism. Treatment of cells with VEGF inhibitors reduced viral loads in tissue culture models as well as reducing lethality in a mouse model of Ebola infection. In this model, the VEGF inhibitors were more effective as prophylactics than as treatments with 50 and 20% reductions in lethality respectively (84). In a second kinome study, investigations performed on human monocytes in response to Monkeypox infection identified phosphorylation events associated with Akt specific to a more pathogenic clade of the virus (Congo Basin MPXV) as compared to Western African MPXV, which has lower rates of lethality. The use of kinase inhibitors to Akt phosphorylation resulted in a significant reduction in viral titres of Congo Basin MPXV but, as predicted by the kinome data, did not impact viral replication of Western African MPXV (85). Given the potential for kinome analysis to rapidly translate into potential therapeutics, including the repurposing of licensed therapeutics, it is not surprising that the technology has been incorporated for characterizing high consequence pathogens (86).

While kinase inhibitors are unlikely to be used in the treatment of livestock, the identification of host responses associated with effective clearance of the pathogen may enable the development of alternative therapeutic approaches, such as vaccines, which are more amenable to livestock. These examples also highlight the potential to apply kinome analysis to animal models of human diseases, or diseases causing co-infection of animals and humans, to the identification of potential human therapies.

## Kinome Analysis of Responses of Livestock to Stress

Livestock are exposed to a multitude of stressors during routine industry practices like weaning, shipping, and restraint. There is a growing appreciation within the livestock industry of the negative consequences of these stresses. Stress decreases milk production, decreases weight gain, and compromises meat quality and increases susceptibility to, and severity of, infectious disease (87). With implications for animal health, well-being and productivity, minimizing animal stress through improved animal management procedures and/or selective breeding is becoming a priority to the livestock industry. Effective management of stress, however, depends on the ability to identify and quantify the effects of various stressors and determine if individual or combined stressors have distinct biological effects.

### Responses of Cattle to Restraint Stress

Cattle are commonly restrained during routine handling practices such as vaccination, therapeutic intervention, and transport. Restraint can elevate plasma cortisol (88), heart rate, and breathing rate (89). As not all animals respond equally to restraint stress, there is a desire to better understand the molecular basis of the stress responses as well as to identify biomarkers that anticipate maladaptive responses to stress.

In an effort to fully describe the range of response to restraint stress, cattle were subject to repeated episodes of brief (5 min) restraint and evaluated for behavioral (chute entry order, chute behavior, and exit velocity), physiological (serum cortisol), and biochemical (kinome) responses. Based on serum cortisol levels (the traditional biomarker of stress responses) sub-groups of animals representing the extremes of stress response were identified. Kinome profiling of PBMCs collected from these animals following a restraint episode revealed distinct signaling events between the high and low cortisol responders. These signaling patterns anticipated differences in apoptosis and carbohydrate metabolism between the two phenotypes, biological differences which were validated through independent techniques (90). In particular, the kinome data anticipated a shift toward the anabolic stage of glycogen metabolism in the high stress responding animals, a finding that was verified by elevated serum glucose levels as well as depleted glycogen stores in the animals of this phenotype. Most importantly, serum glucose provided a reliable, inexpensive indicator of serum cortisol levels and often had greater predictive value than cortisol for stress-related behavioral responses (90).

### Responses of Chicken to Heat Stress

Livestock are often exposed to environmental conditions that impact their health and well-being. For example, during mass transport poultry are subject to conditions that can result in significant fatalities as well as compromising the health and meat quality of the surviving animals (91). Much of this reflects extremes of temperature that can exist within shipping containers, in particular during transport in harsh climates. As a consequence of the positioning of the heating and cooling systems the front of the container often results in temperature extremes at the front and back of the container. Each of these extremes of temperature can have negative consequences on animal health.

To identify the molecular mechanisms underlying these changes, a species-customized peptide array was created for kinome analysis of chickens. This array was designed with a specific priority to include representation of phosphorylation events with central roles in regulation of metabolism. As different regions of the body have unique responses and susceptibilities to thermal stress, kinome analysis was performed on breast and thigh muscle in response to both hot (+35°C) and cold (−15°C) stress temperatures that mimic those which are often experienced during transport. Initial evaluation of meat quality following stress treatments revealed cold stress, compared to heat stress, was more detrimental to meat quality as evident by increased pH_u_, water binding capacity, darker color and lower glycolytic potential – these effects were more pronounced in the thigh than the breast muscle. Subsequent kinome analysis revealed tissue-specific phosphorylation events occurring in breast and thigh muscle (92). Specific to breast muscle, pathway analysis revealed the activation of ErbB signaling pathway in response to cold stress, a pathway implicated as having cytoprotective effects in various animal models and associated with muscle repair and cell survival. This finding was consistent with the moderate effect of thermal stress observed in breast tissue. Conversely, thigh muscle, which showed extensive changes in meat quality following cold stress, resulted in the activation of innate immune response and TGF-β signaling, pathways commonly associated with tissue damage and repair responses. Collectively, this study offers insight into the unique susceptibilities, as well as functional consequences, of thermal stress of these tissues.

A further important outcome of this analysis was the observation that samples from different muscle types had distinct signaling profiles, even in the absence of thermal stress. This contributes to, and adds depth to, the emerging hypothesis that distinct phenotypes are often reflected at the level of the kinome; that distinct signaling profiles (kinotypes) exist across species, between individuals of the same species, and within different tissues of the same individual (93). This was later supported by an investigation of cattle which demonstrated distinct patterns of kinome activity within adjacent, but functionally distinct, regions of the intestine (94). Further to this, comparative kinome analysis of CD21^+^ B cells obtained from two anatomically distinct sites (i.e., intestinal Peyer’s patches and blood) revealed significant differences in signaling profiles as well as offering mechanistic insight into critical functional differences between these populations (94).

## Future Directions

### Technological Limitations (Software)

Even with the development of computational tools customized specifically for kinome data analysis, a few technical challenges still need to be addressed. Kinome arrays can create a large amount of statistical noise, due to a low signal-to-noise ratio, that may interfere with the results. Currently, this is mitigated through the use of normalization techniques such as variance stabilizing normalization, however no technique can remove all statistical noise. Advancements in normalization methods can be integrated into the kinome analysis pipeline but many features need to be specifically tailored to the unique characteristics of kinome data. A number of key sources of noise that affect kinome arrays have been identified.

A large degree of variance can be seen across regions of a peptide microarray. One source of such variance causes increased foreground and background signal at the bottom of the array resulting in spatial bias. This is a systemic bias as a result of uneven distribution of biological sample applied to the array and/or the stain used to bind and detect phosphorylated residues. There are methods that sharply reduce this variance that have been implemented in PIIKA. However, there is location-based variance between the edges and the center of the array, and variance between replicate blocks that currently cannot be corrected for as easily.

While the development of PIIKA has been a large improvement from using DNA microarray technology for kinome data analysis, there remains the necessity for using DNA microarray software for image analysis as no kinome-specific technology has been created to date. This leads to issues regarding the alignment of the spots on the microarray. For now, a large degree of manual alignment is required for kinome arrays that is done relatively automatically for DNA microarrays. This is not only a large investment of time, especially in experiments with a large number of arrays and replicates, it is also subject to error. The outcome of this type of error can be categorized by the shift in the foreground mean relative to the foreground median. This indicates the mislabeling of pixels of the spots as foreground rather than background. This is then improperly corrected and causes the foreground pixels to be asymmetrically distributed as the majority of pixels in an improperly aligned spot are mislabeled, and ultimately results in a foreground mean that is much lower than the actual value. It is very possible that the mean values of many of the top hits are significantly lower than they should be and thereby not appearing in the results as a top hit.

The selection of the top hits in a kinome array must be done carefully, and with the amount of noise often present in the output it is necessary to apply statistical methods to normalize the data and remove background noise. However, different methods have vastly different outcomes, and with a plethora of possible tools, algorithms and techniques to choose from, it is unknown which methods translate more accurately to biological truth. This may not be consistent among different experiments and array conditions. There may be specific characteristics of arrays that favor different statistical methods. Further investigation into which techniques are best applied situationally is needed.

### Technological Limitations (Hardware)

Currently, the majority of peptide array kinome efforts utilize either radioactivity or phosphorylation-specific stains for the detection of the signal on the arrays; neither of these options are ideal. Radioactive approaches are challenged by issues relating to safety and regulation while the stains can be problematic due to lack of specificity, including background staining of the arrays which complicates evaluation of the signal-to-noise ratios. Other detection methods should be investigated. This could include a variety of antibodies with general reactivity toward phosphorylated residues; either reactivity with modified serine and threonine residues, or serine, threonine, and tyrosine, or a combination of such antibodies. There are modified forms of ATP, such as gamma-modified ATP analogs, which are functionally analogous to the radioactive derivatives but with a basis of detection in fluorescent labeling (95). These ATP analogs might be effective for peptide array investigations, although the efficiency by which they are recognized and utilized by the various kinases would need to be carefully defined. At the very least, these alternatives for detection should be evaluated within a comparative experiment.

### Realms of Application

Within the upcoming years there are a number of fields of investigation which would seem logical and strategy directions to apply the kinome, both in terms of organisms to be considered, applications to understanding the mechanisms and efficiencies of treatments, and philosophies of application. While the information provided by DAPPLE2 enables the creation of customized peptide arrays for virtually any species, there are a couple of areas where there seems to particularly promising and strategic opportunities.

### Kinome Analysis for Insects and Other Small Eukaryotes

The use of peptide arrays to define signaling events within the honeybee highlights the potential for the application of this approach to other insect species which are of environmental, economic, and scientific importance. In particular, the opportunity to conduct whole-organism kinome profiling to identify biomarkers and mechanisms of phenotypes could enable high-throughput screening efforts of insects. For example, this additional layer of information could be particularly useful for species, such as *D. melanogaster*, which have been extensively characterized through genomic approaches. As the phosphoproteome of Drosophila has been experimentally defined, creation of a highly reliable Drosophila-specific array would be a straightforward endeavor. The defined phosphoproteome of Drosophila also serves as an important resource for the creation of peptide arrays for other insect species of undefined phosphoproteomes, as was the case for honeybees (96).

In contrast to the distinct, phenotype-specific signaling profiles that were observed in whole organism kinome profiling of honeybees, within higher organisms there is clear evidence for distinct patterns of signaling within different regions of the body, and even within specialized compartments of the same region of the body. However, the lower levels of tissue specialization within smaller organisms may translate into a more homogenous signaling response throughout the organism. It would be interesting to investigate the extent to which whole organism kinome profiling can be effectively applied to other small eukaryotes, in particular those that serve as important research models, such as nematodes, would seem logical targets for future investigations.

### Plants

Considerable efforts have been expended to define both the kinase complement (in terms of number and identities of kinases) and the phosphoproteomes of many plant species (97–99). As with animal species used for food production, infectious diseases and stress are also two major priorities affecting crop production. Kinome analysis could similarly be applied to define phenotypic traits associated with increased stress tolerance and susceptibility, in addition to better understanding plant immune defenses against the diverse range of pathogens they encounter. There has been considerably less effort toward global kinome profiling in plants, in particular through peptide arrays. There are two publications describing the application of peptide arrays to define kinome activity in the model organism *Arabidopsis thaliana* (100, 101). The peptide arrays utilized in these investigations were not customized to reflect the Arabidopsis phosphoproteome. While these investigations supply high-level proof-of-principle evidence of the opportunity to apply the technology to plants, it is very difficult to extract specific biology from this type of cross-species application of peptide arrays. Given the efforts expended to define plant responses through other omic approaches, as well as the success achieved in translating this approach to livestock, it would seem timely and appropriate to extend this approach to plants. In particular, the available plant phosphoproteomes databases can enable the creation of peptide arrays based on experimentally defined phosphorylation sites as well as serving as an effective starting point for the creation of arrays for plant species whose phosphoproteomes have yet to be defined.

## Mechanisms of Therapeutics

In addition to the potential to use kinome analysis to inform rational selection of therapeutics, there may also be opportunities to decipher the mechanisms of action of potential therapeutics. Such an approach could be applied to inform the rational selection, application, and refinement of these treatments. The ability of kinome analysis to offer nuanced information about host responses could also provide valuable correlates of protection to facilitate high-throughput screening of libraries of potential therapeutic agents and/or treatments. Specifically, there is an opportunity to apply kinome to investigate the modes of action for prebiotics, probiotics and postbiotics. There is rapidly growing appreciation of the importance of the microbiome to human and animal health. With this, there is emerging priority to manipulate the commensal bacterial environment through the use of prebiotics, probiotics, and postbiotics. This is not a trivial task. The contributions of the microbiome reflect complex, dynamic interactions between the host and associated microbes. More specific information on the mechanisms and consequences of action of these interactions would enable rational application and refinements of these treatments, including indicators to define therapeutic benefits.

### Prebiotics

Prebiotics are non-digestible sugars that are utilized to promote the establishment of a healthy microbiome. The traditional view is that prebiotics function by promoting the growth of beneficial gut microbes, independent of any direct effects on the host. An investigation was conducted to determine the occurrence and consequences of direct impacts of prebiotics on the intestinal mucosa. Treatment with two commercial prebiotics, inulin and short-chain fructo-oligosaccharide, in the absence of microbes, had beneficial responses by promoting intestinal epithelial integrity to limit barrier disruptions by pathogenic intestinal microbes. These outcomes were achieved through the induction of select tight junction proteins via a mechanism involving activation of protein kinase C signaling (102). In addition to specific information of the action, mechanisms, and consequences by which these prebiotics exert barrier protective effects on the intestinal epithelium, this study also challenged the paradigm that the action of prebiotics was limited to, and dependent on, microbial influence. Shifting this perspective will enable new opportunities for the selection and refinement of prebiotics.

### Postbiotics

A major contributor to the evolution of antibiotic resistance is their overuse as growth-promoters in livestock species such as poultry. Probiotics present an attractive alternative to populate the alimentary tract with beneficial microbes to improve overall health. Postbiotics provide products derived from probiotics to elicit and prime beneficial host immune responses. Kinome analysis was recently used to understand how postbiotics administered to *Clostridium perfringens*-challenged broiler chickens altered the intestinal microenvironment to contribute to reduced lesion scoring, lower bacterial loads and mortality (103). Comparative analysis of intestinal tissue extracts from chickens administered the postbiotic alone showed very few peptide phosphorylation events on chicken species-specific kinome arrays when reacted with duodenal tissue extracts, but extensive kinase-mediated phosphorylation events when reacted with jejunal tissue extracts. Further analysis of jejunal tissue revealed postbiotic treatment alone impacted peptides associated with innate immune pathways. By contrast, jejunal tissue extracts from chickens challenged with *C. perfringens* alone revealed kinase-mediated phosphorylation of peptides associated with T cell receptor signaling, natural killer cell mediated cytotoxicity, and the Fc epsilon receptor I signaling suggesting the induction of adaptive immune pathways. In chickens challenged with *C. perfringens* after receiving the postbiotic treatment pathway analysis revealed, unexpectedly, activation of overall fewer immune-related pathways in jejunal tissue as compared to either postbiotic administration alone or *C. perfringens* challenge alone.

These data suggest that postbiotics induced an immune-modulating effect in the jejunum of broiler chickens resulting in an altered intestinal microenvironment capable of controlling *C. perfringens* infection while, as importantly, maintaining gut barrier and function. Further investigation into this balanced immune response will provide critical information on understanding how mucosal surfaces can control infection while maintaining a homeostatic (i.e., anti-inflammatory) state. Moreover, this study shows that kinome technology can provide a novel, and complementary, approach in understanding the mode of action for these alternative, antibiotic-independent therapeutics.

### Correlates of Immune Protection

Researchers typically focus on a very limited number of correlates of immune protection for screening vaccine antigens. However, chronic infectious diseases, in particular, would benefit from a more comprehensive assessment of immune responses both during infection and in response to vaccines. As an example, there is clear evidence that IFNγ is a key cytokine contributing to control and clearance of mycobacterial infection. However, *in vivo*, IFNγ has not been a reliable correlate of immune protection for either *M. paratuberculosis* in bovine Johne’s disease (104, 105) or *Mycobacterium bovis* in bovine tuberculosis (106). Thus, employing IFNγ as the sole readout of immune protection when screening for potential vaccine candidates could negate a number of promising candidates. Understanding the processes leading to IFNγ induction, and the subsequent consequences, would create a better understanding of immune protection and lead to better selection of immune correlates when screening vaccine antigens.

### Phenotype-Driven Kinome Profiling

Thus far, kinome profiling has typically been applied to describe signaling events in response to a defined stimulus. There is, however, the opportunity to adapt a “phenotype first” approach that has been highly successful for other omic approaches. That is, to identify and investigate phenotypically distinct sub-groups of a population in an effort to identify molecular biomarkers and mechanistic insight into those differences. The example of describing mechanisms of Varroa mite tolerance within colonies of honeybees of distinct phenotypes highlights the potential of this approach for kinome investigations. Within the context of livestock applications such biomarkers could function to guide breeding efforts while in the context of human health applications to differentiate signaling events within healthy and disease-associated individuals.

## Peripheral Blood Mononuclear Cells

The first use of peptide arrays for global kinome profiling was to describe signaling events within human PBMCs in response to *ex vivo* stimulation with LPS (27). Since this foundational work PBMCs have remained a convenient and informative cellular population for describing responses to a variety of stimuli but with a shift from *ex vivo* to *in vivo* stimulations. A critical advantage of PBMCs is the opportunity to collect samples pre- and post-treatment which provides a valuable control to minimize the contributions of the individual-specific kinome profiles. For example, recent efforts for kinome profiling of human PBMCs have investigated responses to acute stress in the form of bungee jumping (107) while another investigation of human PBMCs facilitated investigation of signaling responses activated by consumption of marijuana (108). Important phenotypic differences between individuals, in particular with respect to immune function, may also manifest in unique signaling profiles of PBMCs.

## Integration With Other Omics

As the respective omic disciplines evolve and refine there is the overarching priority for more systems biology perspectives that integrate cellular responses across a range of biomolecular levels. To date there has been minimal effort to attempt to integrate peptide array kinome data with the outputs of other omic approaches, at least on a global scale.

From a more focused perspective, gene expression data has often been used to verify the results of kinome studies. For example, the observation of activation of interferon-associated signaling pathways was shown to coincide with elevated expression of interferon-regulated genes, highlighting the potential for cohesion between transcriptional and kinomic responses. Other examples can highlight a seeming disconnect between transcriptional and kinomic responses; *M. paratuberculosis*-infected bovine monocytes had elevated levels of expression of a receptor but decreased signaling through the associated pathway. The information from each of these techniques is not actually contradictory and these differences instead highlight a key biological mechanism that occurs within the functional realms that separate gene expression and signal transduction. Efforts to merge kinome datasets with those from other omics should look for not only supporting evidence where the same biology is suggested at each level of investigation but also where the results would appear in contradiction with each other may suggest important points of regulation. The implication of the same biology, independent of the implied direction of change, activation or inhibition, nevertheless still implicates the involvement of that process.

## Concluding Remarks

In the decade since the development of the first species-specific peptide array there have been a wealth of publications that demonstrate the value of kinome analysis for investigations of livestock species. There has been a consistent evolution in the tools supporting and enabling kinome analysis which have enabled a consistent progression of the technology and the complexity of the biology which is addressed. The technology has not, however, achieved a point where it has been widely incorporated into research programs. Instead, there is the trend toward relatively small groups of labs applying the technology through collaboration, organized research groups within large organizations, and as fee-for-service opportunities, both by commercial companies as well as units within academia (University of Delaware Kinome Center).

The coming years will likely see the expansion of the peptide arrays into new spheres of application including the characterization of insect and plant species. Within these realms, there will be greater opportunities to apply the technology to samples of defined genetic and phenotypic diversity that will strengthen the relationship between kinotypes and phenotypes.

Ironically, while the initial applications of peptide arrays to livestock were inspired by advances in kinome science within human health realms, the advances which have been made during efforts to apply the technology to livestock are now increasingly being applied within human health applications. This reflects both the advances in the software for analysis of kinome data but also by facilitating the development of peptide arrays that are customized for consideration of specific biology. This is a healthy and desired evolution of the technology as the emergence of kinome data from a variety of species will enable greater opportunities to consider phosphorylation-mediated signal transduction from species-comparative, evolutionary perspectives. This will undoubtedly include emphasis on how the presence and absence of specific kinases and phosphorylation sites across species impact the phenotypic characteristics of the organism that may serve as an important evolutionary selection pressure.

## Author Contributions

All authors contributed to writing and preparing the manuscript.

## Conflict of Interest

The authors declare that the research was conducted in the absence of any commercial or financial relationships that could be construed as a potential conflict of interest.
